# Challenges and Solutions Perceived by Educators in an Early Childcare Program for Refugee Children

**DOI:** 10.3389/fpsyg.2018.01621

**Published:** 2018-09-06

**Authors:** Julian Busch, Lilly-Marlen Bihler, Hanna Lembcke, Thimo Buchmüller, Katerina Diers, Birgit Leyendecker

**Affiliations:** Department of Developmental Psychology, Faculty of Psychology, Ruhr University Bochum, Bochum, Germany

**Keywords:** early childhood education, childcare, refugee, challenges, preschool

## Abstract

Immigration to Germany peaked in 2016. More than 105,000 refugees below the age of 7 years arrived within 12 months. Since then, Germany and other host nations have been in need of strategies to cover the emerging demand for childcare services. The German federal state North-Rhine Westphalia has funded a specialized early childhood education and care (ECEC) program for recently arrived refugees. The present study investigated challenges and possible solutions in this specialized ECEC. In a pilot study, inductive content analysis of *n*_1_ = 28 semi-structured interviews with early childhood educators revealed 19 distinct challenges and four generic categories for solutions (provide clear and predictable structures, involve and support parents, ensure adequate structural features of the childcare group, convey trust and feelings of competence). For the main study, identified challenges were transcribed into items for a closed-format questionnaire, which was distributed to a second sample of educators (*n*_2_ = 96). Challenges perceived as most difficult concerned language barriers and communication with parents. An exploratory factor analysis of the challenges questionnaire yielded four underlying domains (interpersonal stress, feasibility and attendance, cultural and communication barriers, structural features of a childcare group). Our study provides a first basis to adapt childcare settings for refugees, and to guide staff training for this special group. We discuss evidence in regard to understanding how ECEC programs can successfully promote refugee children’s psychosocial adaptation and educational outcomes.

## Introduction

When immigration peaked in 2016, Germany received more than 105,000 applications for asylum from children below the age of 7 years within a 12 months period. Two-thirds originated from Syria, Afghanistan, or Iraq (German Federal Agency for Migration and Refugees, 2016). Refugee children often have disrupted educational biographies. In early childhood, precarious environments potentially jeopardize children’s successful transition into early childcare programs (Sirin and Rogers-Sirin, 2015). Recent immigration, therefore, poses a challenge for policy makers, early childhood educators, and caregivers in Germany. The enrollment rates of recently arrived refugee children in ECEC programs are currently lower compared to those of non-refugee children (Gross and Ntagengwa, 2016; Gambaro et al., 2017). However, the number of arrived children exceeds the current number of available places in German early childhood education and care (ECEC) programs. North-Rhine Westphalia, the largest of all German federal states, hosts more than one quarter of all refugees. Its Federal Ministry for Children, Women, Refugees and Integration responded to this new demand for childcare places by establishing “Bridging Projects" for recently arrived refugee children. The aims of these provisional childcare groups are to compensate for the lack of regular childcare places and to facilitate their subsequent transition into regular ECEC programs. There are some legal restrictions to receive funding for a Bridging Project. At least one educator needs a childcare-related qualification, and the educator-child ratio should be 1:5 or better. Setting, equipment, and schedule of these childcare groups may vary, depending on the context. More than 1,100 diverse childcare groups have been established since May 2015.

Several studies have shown that the degree of social support, community integration, and reinstatement in ECEC programs are associated with psychosocial adjustment and developmental outcomes of refugee children (Sirin and Rogers-Sirin, 2015). ECEC program attendance has also been associated with higher academic performance, better health, and higher rates of later employment in the general population (Schweinhart, 1993), as well as in immigrant, and disadvantaged, populations (Han, 2008; Votruba-Drzal et al., 2015). Despite this evidence, research on how to integrate recently arrived refugee children into ECEC programs is still scarce. When adapting childcare, it is essential that policy makers and educators know about the challenges arising from the transition of refugee children into ECEC programs, and effective solutions. However, educators expressed concern about lacking cultural competence, and reported discomfort when policies conflicted with families’ cultural norms and practices (Hurley et al., 2011). A multi-center study on refugees in social services in the south of the United States showed that refugee families were more likely to choose informal childcare options (Farrell et al., 2008). Reasons for their usage preferences were easier access, accordance with their cultural values, and opportunities for home-language support.

Some studies investigated challenging topics in childcare with refugee children. Hurley et al. (2013) interviewed 25 preschool service providers in New England. The identified themes concerned current life circumstances of refugees (social isolation and resettlement stress), cultural dissonances between educators and families (expectations toward childcare and child rearing practices), and the required competencies of children for early childcare (language and self-regulation). Another interview study with 26 diverse refugee families and educators in the state of New York found the language barrier to be a distinct demand (Szente et al., 2006). Other identified challenges in this study were related to structural features, and to feasibility of ECEC with refugee children. For a German childcare group with refugees from the Roma community, Hahn (2011) found challenges concerning adequate equipment, continuous funding, and trained personal. In recent field visits of Bridging Projects our research team moreover learned about infrequent attendance and fluctuation of refugee children due to deportation as further obstacles in the stable integration of them into early childcare (Busch et al., unpublished).

There is less evidence about solutions for the specific challenges in childcare with refugee children. Some studies focus on how educators can support refugee children as they cope with emotional problems and psychosocial adjustment. Findings from an ethnographic case study in an early childcare group with refugee children in Norway (Kalkman and Clark, 2017) suggested beneficial effects of role-play activities. They argued that role-play facilitates the reprocessing of past events and fosters the ability to recognize social activities, cultural identity, and local traditions. Consistent to this approach, researchers in Canada examined the “sand-play program” for emotional problems of 4- to 5-year-old refugee and immigrant children in childcare groups in a randomized and controlled effectiveness study. The researchers concluded that children expressed and processed their emotions through play behavior as they made references to past experiences (Lacroix et al., 2007). Repeated assessments by parents and educators showed that the sand-play program reduced psychological stress (Rousseau et al., 2009).

Further studies focused on effective pathways to integrate refugee families into childcare, and to adapt services correspondingly. In his report, Waniganayake (2001) recommended strategies for successfully working with refugees in childcare, such as providing children with opportunities for expression, setting clear boundaries, teaching alternative conflict resolution strategies, or visiting refugee families at home. In the interview study by Hurley et al. (2013), educators in childcare with refugees suggested considering community food preparation to bond with the parents, providing routines to make children feel more comfortable, and using pictures and symbols to express emotions. Poureslami et al. (2013) utilized focus groups with childcare providers, educators, and immigrant parents to identify five domains to promote the transition of refugee children into childcare. These domains were a centralized system (linking existing programs and sharing expertise on different cultural communities), support of childcare staff (dealing with cultural diversity and strategies to introduce new participants), effective announcement of services (in schools, communities, and media), educational materials (information for parents), and program structure (flexible operating hours, transport, and parental involvement).

It is still unclear, which theoretical approach geared at educators in ECEC programs can guide research and inform us about challenges and solutions with recently arrived refugee children. In order to systematically structure challenges and solutions in ECEC programs with refugee children, Hurley et al. (2013) discussed the pyramid model (Fox et al., 2003). This three-tiered model aims to foster positive socio-emotional and behavioral developmental outcomes. The first tier, “universal promotions,” provides nurturing environments and stimulation to every child in a childcare group in order to foster their development and acquisition of competencies. The second tier focuses on the special needs of certain groups of children, i.e., refugee children. Secondary prevention and intervention strategies are employed which address needs related to the educational gap, resettlement, and culture of refugee children, and may directly foster the required competencies (e.g., language and self-regulation) and psychosocial adjustment. The third tier contains tertiary interventions that are often administered by a multi-professional team according to an individual, intensive support plan. In the case of childcare for recently arrived refugees, third tier intervention may encompass professional trauma therapy, as well as practical and holistic support to overcome obstacles during resettlement.

To date, educators can rely on few research-based experiences in childcare with refugee children (Tadesse et al., 2009). However, they are confronted with several specific challenges in the transition of refugee children into ECEC programs. Scientific evidence on this topic is limited from a geographical, methodological, and conceptual perspective. Geographically, available studies were predominantly conducted in countries with extensive resettlement programs and under specific policies (e.g., Canada, United States, and Australia), whereas research from European countries is very scarce. Methodologically, evidence in this research field builds less on analytic studies, and mainly on small sample sizes (i.e., case reports and case series), and expert opinions. From a conceptual perspective, few findings on childcare with refugee children were organized according to a theoretical model. Research on ECEC programs with refugee children needs to overcome these limitations in order to obtain valid and generalizable findings. The aim of the present study was to investigate challenges and solutions in the Bridging Projects, as perceived by the educators. This research was divided into a pilot study and a main study. The pilot study investigated challenges and possible solutions using a qualitative approach. For the main study, a closed-ended questionnaire was created based on data from the pilot study. The questionnaire was used to assess the severity of certain challenges and to systemize them by applying factor analysis. Findings from both study parts will be integrated in the general discussion.

## Pilot Study

### Methods

#### Participants

Our research team visited a total number of 50 Bridging Projects for field observations. One educator, from each respective 28 Bridging Project, participated in the pilot study during or subsequent to the visit. These educators on average were 42 years old (*SD*_age_ = 11.24 years). All but two were female. On average, the 28 Bridging Projects were attended by 9 refugee children (*SD*_number of children_ = 4.50, range_number of children_ = 1–15).

#### Material

We provided a paper-pencil survey to educators in Bridging Projects. They answered two open format questions in writing: (1) “What are specific challenges in the work with refugee children?” and (2) “What are proven or possible solutions concerning these challenges?” Additionally, educators reported socio-demographic information about themselves.

#### Data Analysis

Four research assistants, two with a master’s and two with a bachelor’s degree in psychology, established a focus group. Based on the procedure described by Elo and Kyngäs (2008), the focus group conducted inductive content analysis. Generic categories were individually generated through open coding of raw responses, and repeatedly discussed. To check reliability of obtained categorization, two research assistants independently assigned the open responses of educators to the generic categories for perceived challenges and solutions, respectively.

### Results

We analyzed responses of all *n*_1_ = 28 educators for the perceived challenges in childcare settings for refugees, and *n*_2_ = 18 responses for possible solutions. A *post hoc* reliability check revealed an overall moderate interrater reliability (challenges: *Kappa* = 0.52, *p* < 0.001; solutions: *Kappa* = 0.59, *p* < 0.001; Landis and Koch, 1977). Perceived challenges were arranged into six generic categories by the focus group (see **Table 1**). Educators mentioned organizational (e.g., accessibility), interpersonal (e.g., communication, behavior, and conflict), and cultural topics (e.g., parenting and roles) as challenges. Considering potential solutions, the focus group identified four generic categories in inductive content analysis, which are shown in **Table 2**. Mentioned solutions concerned different qualities of childcare, i.e., process quality (e.g., convey trust and competence), structural quality (sufficient material), and childcare group conception (session structuring and parental involvement).

**Table 1:** Description of generic categories for perceived challenges.

Generic category	Description
Communication	- Language barriers.
	- Dealing with hot topics (e.g., wearing a head scarf).
	- Establishing cohesion in a linguistically diverse group.
	- Parents need more time to understand routines.
Child behavior	- Behavior problems (e.g., anxiety, withdrawal, restlessness, emotional outbursts).
	- Children re-enact war-scenes.
	- Tardiness.
	- Reliability and infrequent attendance of refugees.
Interpersonal conflicts	- Caregivers’ expectations toward childcare.
	- Conflicts between children (e.g., teasing); children and educators (e.g., children break the rules frequently, children have difficulties regulating closeness-distance); educators and parents (e.g., the parents do not intervene at misbehavior of own children); participating parents (e.g., between nationalities).
Flight-related experiences	- Sensitive topics strain educators emotionally (e.g., asylum, deportation, war-experience, separation of families).
	- Educators lack knowledge about the experiences and biographies of refugees.
	- Educators struggle empathizing with refugees.
Structural features of a childcare Group	- Accessibility of childcare groups (e.g., connection to public transport).
	- Insufficient educator-child ratio.
	- Group fluctuation.
	- Insufficient equipment (e.g., too many donated toys).
	- Insufficient settings for childcare.
	- Age differences between attending children.
Intercultural understanding	- Differences in parenting, culture, traditions.
	- Social relations between actors in childcare (e.g., role of educators).

*Generic categories and descriptions for solutions derived from inductive content analysis (n_educators_ = 28).*

**Table 2:** Description of generic categories for solution approaches.

Generic category	Description
Provide clear and predictable structures	- Starting every day with a morning circle.
	- Communicate and enforce rules in a comprehensible way.

Involve and support parents	- Establishing new rules in consultation with parents.
	- Provide support, even for other areas of life.

Ensure adequate structural features of the childcare group	- Improve educator-child ratio (allowing one-on-one care, if necessary).
	- Provide appropriate material (e.g., games to learn self regulation, language).
	- Provide material that is easy to understand.

Convey trust and feelings of competence	- Make children feel welcome.
	- Be kind, reliable, and trustworthy.
	- Recognize children’s talents.
	- Give children enough time for integration.

*Generic categories and descriptions for solutions derived from inductive content analysis (n_educators_ = 18).*

### Discussion

Educators report different topics as challenging in the Bridging Projects. Obtained information was aggregated into six generic categories by a focus group according to similarities in educators’ responses. We added new evidence to this understudied field of research by informing about challenges in childcare settings for refugee children. However, there are shortcomings in the approach of relying on openly reported experiences of educators. We only received responses from 28 Bridging Projects, which seems insufficient to conclude generalizability of the challenges. We moreover do neither know about the significance of each of those challenges nor the validity of the generic categories.

Secondly, our pilot study identified potential solutions to the perceived challenges. Only 18 educators responded to this open-format question. Several educators in the Bridging Projects might have limited experiences with refugee families yet and therefore omitted this question. If this conclusion based on educators’ response behavior is valid, it hints to a strong need for new strategies in childcare settings for refugees. Nevertheless, obtained answers reveal first insights into current childcare practices with refugees in specialized ECEC programs from an educator’s perspective. Findings on potential solutions are preliminary and need further empirical evidence. Linking those preliminary findings on solutions with empirically validated findings on challenges in a theoretical framework could increase applicability of our evidence for childcare practice.

## Main Study

### Methods

#### Participants

For the main study, we randomly contacted a second sample of Bridging Projects via email and telephone. One educator per Bridging Project was asked to complete an online questionnaire about the perceived challenges. Overall, 96 educators (*M*_age_ = 43.48 years, *SD*_age_ = 11.83 years; 91% female) participated in the survey. On average, 10 children *(SD*_number of children_ = 8.58, *range*_number of children_ = 2–60) attended each of these Bridging Projects on a regular basis.

#### Instruments

The aforementioned focus group created items for the closed-ended questionnaire. In a first step, the group members generated items on potential challenges via open coding of the responses received by educators in the pilot study. Each member was asked to directly segment and code the educators’ open responses. Creating items directly from the pure data (instead of the content-analysis from pilot study) limited circularity when comparing qualitative and quantitative results of both study parts. In a second step, the focus group selected a set of 19 items during group conversation. The rationale for the selection of items was to ensure that the scope of educators’ open responses would be covered. Responses to the questionnaire were given on a five-point Likert scale, which was presented with three anchors (1 = not challenging at all, 3 = somewhat challenging, 5 = very challenging).

#### Data Analysis

We calculated the mean and standard deviation for each item of the questionnaire. The descriptive analysis allows the ranking of distinct challenges across all Bridging Projects. We conducted an exploratory factor analysis with principal axis factoring (EFA; Costello and Osborne, 2005) on the questionnaire in order to validate and extend findings from inductive content analysis. Conceptually, EFA and inductive content analysis both exploratively aggregate data to find domains of higher order. However, a larger sample size for EFA fosters generalizability. The statistical approach of EFA, moreover, is less dependent on the subjectivity of a few coders. EFA goes beyond a content-directed structuring, which exclusively guides inductive content analysis.

For the rotation of the factors in EFA, we used the oblique rotation method oblimin. This non-orthogonal approach has fewer model-restrictions. It is therefore best suited to delineate first evidence in a field. In preliminary analyses, we scanned the correlation matrix for variables that did not correlate, or correlated very highly (>0.90) with any other variable. The overall and variable specific Measures of Sampling Adequacy (MSA) were additionally calculated. *MSA* ≥ 0.50 indicates that the sample size is sufficient to yield distinct and reliable factors in an EFA (Kaiser, 1974). After preliminary analyses, we assessed a reasonable number of factors using Kaiser’s criterion (eigenvalue >1), scree plot examination, and parallel analysis. Overall model fit was examined by the model chi-squared test on an alpha-level of 0.05. Additionally, we considered the root mean square error of approximation (RMSEA), and the Tucker-Lewis index (TLI) for model fit. Good fit was defined by a *RMSEA* ≤ 0.06 and a *TLI* ≥ 0.95 (Hu and Bentler, 1999). Factor loadings ≥0.30 were considered as substantial. The focus group interpreted the factors of challenges from EFA according to the grouping of the items on the latent factors. All statistical analyses were performed in R 3.4.1 using default packages and the “psych” package for EFA.

### Results

#### Hierarchy of Perceived Challenges

The ranking of the challenges according to their ratings is shown in **Table 3**. Educators perceived the language barrier and communication with parents as most challenging. Although mentioned, conflicts and accessibility of a childcare group were perceived as the least severe challenges.

**Table 3:** Factor loadings for EFA with oblimin rotation using all items of the challenges questionnaire.

Item	Factor 1	Factor 2	Factor 3	Factor 4
	Interpersonal stress	Feasibility and attendance	Cultural and communication barriers	Structural features of a childcare group
Conflicts between parents	**0.72**	0.19	−0.09	−0.08
Conflicts between children	**0.68**	−0.26	−0.02	0.19
Conflicts between educators and parents	**0.63**	0.01	0.08	−0.04
Behavior problems of children	**0.51**	−0.08	0.11	0.12
Educators’ emotional stress due to living conditions and flight related experiences of children	**0.47**	0.23	−0.03	0.08
High fluctuation complicates ability to plan	−0.10	**0.80**	−0.08	0.13
Children take part irregularly	0.17	**0.66**	0.09	−0.13
Decreasing number of participants throughout the project	0.01	**0.58**	0.02	0.16
Tardy arrival *(reversed)*	−**0.35**	−**0.40**	−0.04	−0.08
Long-term feasibility of the childcare group	0.11	**0.30**	0.13	−0.07
Linguistic barriers between educators and parents	−0.17	−0.09	**0.80**	0.06
Communication with parents	0.07	0.14	**0.66**	−0.07
Cross-cultural communication barriers	**0.34**	−0.05	**0.53**	−0.02
Parents’ expectations	0.04	−0.03	**0.46**	0.10
Communication barriers between children	0.23	0.23	**0.39**	0.07
Different ages of children	−0.05	0.15	0.12	**0.65**
Educator-child ratio	0.15	0.06	−0.08	**0.54**
Material resources	0.02	−0.08	0.04	**0.48**
Accessible by public transportation	0.26	0.07	−0.05	**0.40**
Eigenvalue (variance explained)	2.48 (13%)	2.03 (11%)	1.83 (10%)	1.37 (7%)

*Questionnaire about the challenges in Bridging Projects; factor loadings ≥0.30 are in boldface; items “cross-cultural communication barriers,” Tardy Arrival revealed cross-loadings with the first factor; EFA, Exploratory factor analysis.*

#### Pre-analysis of EFA

Inspection of the correlation matrix yielded that all variables did correlate with at least one other variable, with no correlation being greater than *r* = 0.90. The overall MSA of 0.67 was satisfactory. The calculation of the variable-specific MSAs indicated that all items reached the critical score of *MSA* ≥ 0.50 (**Table 3**), with an exception for the item “material resources” (*MSA* = 0.39). This indicates that partial correlations of this certain item are low. We decided not to exclude the item in order to not drop reported challenges in the explorative statistical analyses. Scree plot, and parallel analysis indicated that four factors should be retained. **Table 4** shows the obtained factor loadings on each factor. Cross-loadings above the defined threshold emerged for the items “tardy arrival” and “cross-cultural communication barriers.” Considering the overall model fit for the four-factor solution, the chi-squared test was marginally significant [χ^2^_(101)_ = 126.06, *p* = 0.046], the *RMSEA* = 0.064 [0.007, 0.078], and the *TLI* = 0.88. Although the defined criteria for good model fit were not fully met, model parameters indicate that explorative analysis may have led to solid results.

**Table 4:** Hierarchy of challenges in early childcare groups with refugees according to the questionnaire.

Rank	Item	*M*	*Md*	*SD*	*n*	MSA
1	Linguistic barriers between educators and parents	3.96	4	1.02	95	0.51
2	Communication with parents	3.88	4	1.12	93	0.54
3	Long-term feasibility of the childcare group	3.44	3	1.24	93	0.54
4	Tardy arrival *(reversed)*	3.27	3	1.34	92	0.80
5	Behavior problems of children	3.21	3	1.04	94	0.66
6	Children take part irregularly	3.02	3	1.18	94	0.66
7	High fluctuation complicates ability to plan	2.79	3	1.32	95	0.59
8	Different ages of children	2.75	3	1.32	92	0.64
9	Cross-cultural communication barriers	2.69	3	1.05	94	0.74
10	Educators’ emotional stress due to living conditions, and flight related experiences of children	2.55	2.5	1.20	94	0.67
11	Decreasing number of participants throughout the project	2.48	2	1.23	92	0.71
12	Communication barriers between children	2.41	2	0.94	94	0.72
13	Educator-child ratio	2.38	2	1.23	93	0.64
14	Conflicts between children	2.35	2	1.11	93	0.75
15	Parents’ expectations	2.34	2	1.17	93	0.80
16	Material resources	2.08	2	1.07	92	0.39
17	Accessible by public transportation	1.96	2	1.18	89	0.70
18	Conflicts between parents	1.62	1	0.97	93	0.80
19	Conflicts between educators and parents	1.56	1	0.87	94	0.71

*Challenges of the generated questionnaire are displayed and ranked according to their arithmetic mean. Each challenge was rated on a Likert-scale ranging from 1 (not challenging at all) to 5 (very challenging). M, arithmetic mean; Md, median; SD, standard deviation; n, number of educators answering the respective question; MSA, measurement of sampling adequacy.*

#### Domains of EFA

According to the statistical grouping of items, the focus group interpreted and labeled the revealed factors as the following domains of challenges (given in descending order regarding their eigenvalue): Interpersonal Stress (i.e., conflict and behavior problems), Feasibility and Attendance” (i.e., family’s reliability and maintenance of the project), Cultural and Communication Barriers (i.e., exchanging information and dissonance on childcare goals), and Structural Features of a Project” (i.e., ensure functional project and group characteristics). Intercorrelations between factors are displayed in **Table 5**.

**Table 5:** Intercorrelations among factors from EFA.

Factor	1	2	3
1. Interpersonal stress	–		
2. Feasibility and attendance	0.20	–	
3. Cultural and communication barriers	0.11	−0.01	–
4. Structural features of a childcare group	0.18	0.19	0.14

*Pearson’s product-moment intercorrelations between factors of oblique exploratory factor analysis are presented.*

### Discussion

The findings of the main study validate and extend the results from the pilot study. Item ranks inform about what educators perceive as most challenging in childcare settings with refugee children. Those results can guide stakeholders and staff to anticipate and react to potential difficulties during planning and conducting ECEC services with refugee children. Using a statistical approach, EFA systematically validated and systemized findings from the inductive content analysis based on a larger sample. EFA explored which challenges tended to coincide among Bridging Projects irrespective of content relations. Pre-analysis for EFA suggested that the item “material resources” is problematic in this approach. This may due to technical reasons, e.g., a relatively small sample size, an item to subject ratio around 1:5, or vague wording. Alternatively, the item may not demonstrate additional value and should thus be excluded in further conclusive analyses, e.g., scale construction. We kept this item in our explorative investigation, because material resources seemed to be an important issue in improvised Bridging Projects (Busch et al., unpublished).

A comparison of EFA with inductive content analysis generally substantiated validity of the preliminary findings from the pilot study. The generic categories Child Behavior, Interpersonal Conflicts, and Flight-related Experiences from inductive content analysis were subsumed under the latent factor Interpersonal Stress. Generic categories Communication and Intercultural Understanding were reflected in the latent factor Cultural and Communication Barriers. The generic category Structural Features of a Childcare Group remained unchanged. A new latent factor, Feasibility, and Attendance, emerged from the EFA of the main study. This factor was not previously identified in the inductive content analysis of the pilot study. Changes of the factor structure and labeling in EFA seemed to better fit the educators’ perspective. These results are, therefore, potentially better suited to guide effective prevention and intervention strategies.

## General Discussion

Young refugee children are likely to stay in host countries, at least for some years, due to on-going crises in several countries. Thus, the host countries require evidence-based strategies for the transition of arriving children into childcare services. Our study adds systematic evidence to this field by identifying specific challenges that educators perceived in specialized ECEC programs for refugee children. In the pilot study, educators freely reported on challenges and solutions in childcare settings for refugee children. We grouped those challenges and solutions into six and four generic categories, respectively. In the main study, educators ranked distinct challenges, which were derived from the pilot study. We then aggregated the distinct challenges into four higher-order domains. The general discussion of our findings is organized according to those domains. Findings on the solutions are subsequently discussed with respect to the pyramid model.

### Challenge: Interpersonal Stress

Our evidence suggests that educators experience conflict with refugee children, as well as their parents, and is directly confronted with the psychosocial needs of refugee families. Consequently, some educators struggled to cope with the fate of refugee families who expected deportation, experienced recent bereavement, or other psychosocial hardships. Our evidence corresponds to findings on mental health of refugee children. Refugee children in preschool age are at risk for increased levels of anxiety, withdrawal, anger, and emotional outbursts (Bronstein and Montgomery, 2011; Buchmüller et al., 2018). Moreover, refugee children may become confused if rules and childcare practices between home and childcare contexts diverge (Whitmarsh, 2011). Vandenbroeck et al. (2009) interpreted this dissonance as a potential source of conflict. Md-Yunus (2009) reported that educators often struggle to create a culture-sensitive environment in childcare settings. Moreover, educators in German childcare settings reported a demand for assistance with psychological problems of young refugee children (Riedel and Lüders, 2016).

### Challenge: Feasibility and Attendance of Refugee Families

Another domain of challenge in childcare with refugee children subsumes their infrequent attendance, high group fluctuation, and the initial unreliability of some refugee families. As a possible explanation, it is proposed that high levels of mental distress of refugees and low perceptions of self-agency (Mitchell and Ouko, 2012) might disturb parental engagement. Cultural differences in educational aspirations and childcare practices additionally aggravate the extent of parental engagement (Tadesse, 2014). Moreover, some refugee parents struggle with separation from their young children without the support of close relatives, particularly in a foreign context after adverse circumstances (Riedel and Lüders, 2016). Improvised childcare settings, high fluctuation of refugees, and changing premises (i.e., closing of central refugee accommodations) may affect long-term feasibility of Bridging Projects. Educators reported these challenges in our study, yet these specialized childcare groups are considered to be a flexible, temporary service until transition into regular ECEC programs is possible.

### Challenge: Cultural and Communication Barriers

Language and cultural barriers hinder successful communication. Educators in our study perceived these barriers as major obstacles in early childcare programs for refugee families. However, a panacea-like strategy for effective communication seems unrealistic, as refugee families are demographically and ethno-culturally diverse. Regarding the cultural barriers, educators reported to experience different expectations for childcare practices from parents, which represents an additional challenge. ECEC programs similar to Western models of institutionalized childcare are less widespread in several countries from which refugees originate (Mitchell and Ouko, 2012; Poureslami et al., 2013). In a study with 199 educators, Bernhard et al. (1998) reported that a substantial number of refugee parents did not understand the goals of ECEC programs in Canada. Specifically, some studies found refugee parents from different African countries to have their own expectations toward the parent-educator relationship and the format of caregiving, which were different to Western ECEC practices (Tadesse et al., 2009; Whitmarsh, 2011; Tadesse, 2014). Overall, our evidence suggests a need for effective communication, and sensitive strategies to convey intentions and practices of existing ECEC programs in the host country to refugee families. At the same time, ECEC programs need to consider prior experiences those families might have had with childcare in home-countries or during flight.

### Challenge: Structural Features of a Childcare Group

Challenges regarding structural features concerned the large age-range of refugee children, who attended the Bridging Projects, the lack of good educator-child ratios, inadequate premises, lack of equipment for a childcare group, and lack of accessibility of the childcare programs. Responses may to some extent reflect the diversity of Bridging Projects. Systematic field observations suggested that differences in the structural quality of Bridging Projects depend on the childcare setting (Busch et al., unpublished). Moreover, educators reported accessibility of childcare programs by refugees as challenging. Consistently, refugee families in Canada reported a lack of locally available childcare groups (Morantz et al., 2012; Poureslami et al., 2013). In conclusion, stakeholders need to deliberately settle childcare services for refugees to accessible locations, which also meet sufficient structural quality standards.

### Possible Solutions for the Challenges According to the Pyramid Model

The pyramid model (Fox et al., 2003) scaffolds linkages between challenges in childcare settings for refugee children and potential solutions. The first tier of the model subsumes measures for providing a nurturing, need-oriented environment for all children of a group, which facilitates learning experiences and builds positive relationships with participating families. Therefore, the childcare environment must also consider the needs of refugee children. Educators in our study remarked on the scarcity of suited materials for early language learners. They emphasized the importance of easy accessibility for refugee families, and an adequate educator-child ratio. They suggested predictable and reliable structures (e.g., repeating timetables) with consistently enforced rules, which promote routines, and foster reliable relationships with refugee children and parents. Correspondingly, Lunneblad (2017) found that refugee children and caregivers often need more time to familiarize themselves with a childcare setting. Educators in our study suggested that conveying warmth, encouragements, and patience are particularly important to foster positive relationships with refugee children and their families.

The second tier intends to specifically address challenges with refugee children and their families, if necessary. According to the educators in our investigation, additional measures for refugees should target the psychosocial situation of refugees, bonding with parents, and facilitation of communication. In addition to the use interpreters, educators suggested communication-supportive materials, e.g., pictograms or posters, as helpful to overcome the language barrier in early childcare with refugees. In line with our evidence on discrepant views on childcare, Tadesse (2014) reported that some refugee parents show resistance when accepting additional measures in ECEC for their children. Exploration of their individual attitudes toward childcare, provision of information about childcare systems, and educational goals might promote acceptance, and foster parental engagement. Besides potential topics of conflict, educators mentioned the unsteady attendance and tardiness of refugee children as challenging. Therefore, educators suggested drop-off and pick-up services. This could promote the regular attendance of refugees because many families often have scarce resources, or they are not familiar with the use of public transport.

The third tier addresses intensively individualized interventions for some children and families. Refugee children in the Bridging Project groups seem to have specific patterns of mental distress (Buchmüller et al., 2018). The majority of refugee children exhibit mild behavior problems. Some children, however, show severe anxiety and withdrawal behavior. This suggests that, at times, educators must cope with severe emotional problems of refugee children. Educators in our study reported that they were insufficiently prepared to deal with this challenge. Cultural and language barriers hinder sensitive and specific identification of the refugee children in need with available diagnostic tools (Hurley et al., 2014). Training of the educators should, therefore, cover an in-depth understanding about psychosocial symptom manifestations, and specific risk factors for negative developmental outcomes. However, educators should not provide psychological therapy, but detect and refer children in need of intervention to treatment facilities. Close cooperation with communal youth welfare, or health care agencies, might help to conduct intensive psychosocial interventions for the complex, and entangled problems that refugee children and their families face during the post-migration period (Szente et al., 2006; Poureslami et al., 2013). Educators in our study reported that the provision of individual assistance for refugee families beyond childcare, e.g., with obstacles of resettlement, promotes successful conductance of childcare with refugee families.

The pyramid model structures challenges and solutions in childcare with refugee children. However, boundaries between the different tiers follow a theoretical concept, and the evidence is ordered in theoretical accordance. Beyond this theoretical model, the practical feasibility of tiered interventions for refugee children needs additional consideration, as measures are implemented into policy and practice.

### Strengths, Limitations, and Future Research

We were the first research group, who used a stepwise approach to investigate perceived challenges and possible solutions in a specialized ECEC program for refugee children. In the pilot study, a focus group applied inductive content analysis in order to generate a questionnaire on challenges, and to obtain findings on potential solutions. Despite the standardized procedure, personal narratives, and past experiences of coders might, nevertheless, have had an impact on how generic categories and the questionnaire were generated. The main study builds on the closed questionnaire on challenges. Future research should particularly investigate its reliability, validity, and comprehensiveness. Psychometric criteria of our questionnaire seem overall sufficient for EFA, and led to solid factors (Costello and Osborne, 2005; Bühner, 2011). However, the item with low MSA and mediocre subject to item ratio might jeopardize a reliable factor structure.

Current childcare services are in need of research on refugees, with short-term practical impact. The main goal of this study was, therefore, to investigate challenges and solutions in specialized ECEC programs for refugee children. Our findings are consistent with a qualitative study on childcare with refugee families in Canada (Poureslami et al., 2013). Our study extends evidence by methodological triangulation in a larger sample from specialized ECEC programs in Germany. General challenges and solutions for all types of childcare settings for refugees exist along with very specific ones that may vary depending on context, group settings, inclusive or exclusive orientation, and specific childcare goals. Linking type and severity of specific challenges to an ECEC program’s structural characteristics helps to further systematize and evaluate the advantages and obstacles of diverse concepts of childcare settings for refugee children. The newly constructed questionnaire of this study provides a basic tool for such investigations. Besides, our investigation is limited to educators’ perspectives. The perspective of refugee parents might possibly reveal more challenges and solutions in ECEC programs.

To date, the effects of childcare attendance on the psychosocial adjustment, academic achievement, and developmental outcomes of refugee children have been insufficiently studied. We do not yet have substantial evidence on effective pathways for recently arrived refugee children into childcare services, or on solutions to tackle the specific challenges in childcare settings for refugee children.

## Conclusion

Our study contributes to systematic research on childcare services for children. We disentangled and systematized challenges, and possible solutions that educators perceived in specialized ECEC programs for refugee children. We generated a questionnaire on perceived challenges, and embedded our findings into a theory-driven framework on a broader empirical basis. Findings can directly inform educators, stakeholders, and policy makers about the specific challenges of refugee children in early childcare and steps toward effective solutions.

## Ethics Statement

This study was conducted in accordance with the ethical guidelines of the German Psychological Society. The Ethics Committee of the Faculty of Psychology at the Ruhr University Bochum approved the study protocol. All subjects gave written informed consent in accordance with the Declaration of Helsinki.

## Disclosure Statement

The funders had no role in study design, data collection, or analysis, decision to publish or preparation of the manuscript.

## Author Contributions

JB contributed in manuscript writing, data analysis, data interpretation, and study conceptualization. L-MB contributed in data analysis, methodological realization, and manuscript writing. HL contributed in manuscript feedback and revision, discussion of findings, and manuscript writing. TB contributed in study conceptualization and manuscript feedback. KD contributed in data analysis and literature research. BL contributed in manuscript feedback and revision, discussion of findings, and head of the project.

## Conflict of Interest Statement

The authors declare that the research was conducted in the absence of any commercial or financial relationships that could be construed as a potential conflict of interest.
